# Measurement of Human Gait Symmetry using Body Surface Normals Extracted from Depth Maps

**DOI:** 10.3390/s19040891

**Published:** 2019-02-21

**Authors:** Trong-Nguyen Nguyen, Huu-Hung Huynh, Jean Meunier

**Affiliations:** 1 DIRO, University of Montreal, Montreal, QC H3T 1J4, Canada; meunier@iro.umontreal.ca; 2 ITF, The University of Danang—University of Science and Technology, Danang 556361, Vietnam; hhhung@dut.udn.vn

**Keywords:** symmetry, depth map, gait, normal vector, point cloud

## Abstract

In this paper, we introduce an approach for measuring human gait symmetry where the input is a sequence of depth maps of subject walking on a treadmill. Body surface normals are used to describe 3D information of the walking subject in each frame. Two different schemes for embedding the temporal factor into a symmetry index are proposed. Experiments on the whole body, as well as the lower limbs, were also considered to assess the usefulness of upper body information in this task. The potential of our method was demonstrated with a dataset of 97,200 depth maps of nine different walking gaits. An ROC analysis for abnormal gait detection gave the best result (AUC=0.958) compared with other related studies. The experimental results provided by our method confirm the contribution of upper body in gait analysis as well as the reliability of approximating average gait symmetry index without explicitly considering individual gait cycles for asymmetry detection.

## 1. Introduction

Gait analysis has shown a lot of evidences demonstrating its potential to identify and diagnose early neurological and non-neurological musculoskeletal disorders. Gait symmetry is one of the most popular features used to perform these health-related assessments. It is a good indicator of human motion ability to identify pathology and assess recovery for people with asymmetric gait of various origins such as cerebral palsy, stroke, hip or knee arthritis and surgery or leg length discrepancy [1,2,3,4]. Researchers have dealt with the problem of gait symmetry estimation according to various input data types. For instance, the use of body-mounted devices such as inertial sensors [5] or motion capture markers [6] has provided precise measurements and promising results. In this paper, we propose an alternative approach estimating an index of gait symmetry using a vision system. Compared with the mentioned methods, our system does not require the precise positioning of sensors/markers on patient’s body. Besides, our input is acquired from a single camera, the method thus, does not need a functionality of run-time calibration/synchronization as for multiple sensors. Our configuration consists of a treadmill where a patient walks and a depth camera placed in front of it capturing a sequence of depth maps. To avoid occlusions in the depth maps, the treadmill console, handlebars and vertical supports are lowered and placed on the floor in front of the treadmill. The objective of our method is to measure a reasonable index indicating human gait symmetry during a walk. This may work as a patient screening tool providing relevant gait information during a treatment or recovery after a surgery. In addition, the study can be considered as an exploratory examination of surface normals to assess gait asymmetry since this factor plays the principal role in our gait description.

The remaining of this paper is organized as follows: Section 2 provides an overview of related vision-based studies working on human gait symmetry measurement; Section 3 describes our geometric feature that represents informative gait properties, Section 4 gives the schemes of symmetry measurement, Section 5 presents our evaluation and a comparison with related studies using a dataset of multiple data types, and Section 6 gives the final conclusion.

## 2. Related Work

Considering vision-based approaches that measure human gait symmetry, many studies working with low-cost depth cameras have been proposed. These 3D vision systems can reduce the need of manual intervention and then simplify their operation. There are two data types that are popularly employed to represent the 3D walking posture: 3D skeleton and depth map. Typically, a skeleton is directly estimated from the corresponding depth map [7,8] and is represented as a collection of 3D coordinates of body joints. Although this has been applied in many applications (e.g., abnormal gait detection [9] or action recognition [10]), the skeleton estimation is unstable between consecutive frames due to noisy depth maps. Besides, skeleton estimation algorithms usually encounter problems (i.e., provide deformed results) when working with pathological gaits [11]. Taking these factors into account, a sequence of raw depth maps was considered as the input of our processing.

Auvinet et al. [11] proposed a method estimating gait asymmetry based on a depth region of lower limbs of subjects walking on a treadmill. They introduced a Mean Gait Cycle Model (MGCM), that is composed of a sequence of Mean Depth Images (MDIs) for each leg, to obtain a representative step cycle and decrease the influence of noise. This requires gait cycle detection and the registration of depth maps prior to averaging since the subject’s position varies on the treadmill. A gait asymmetry index was measured as the longitudinal difference between (left and right) pairs of such MDIs. Unlike that approach where only the leg zone was considered, the whole depth body is involved into the stage of feature extraction in our method. Besides, our processing flow does not require gait cycle detection since our gait symmetry index can be directly measured on an input sequence of an arbitrary length. Moreover, we are using surface normals that are independent of the body’s position on the treadmill, no registration of depth maps is thus, required.

Another recent method that computes a gait normality index was described in [12]. The authors individually processed the depth and silhouette information to provide a pair of scores that are then weighted summed to get the gait normality index. Depth information was provided by a histogram of depth-related features of keypoints. A hidden Markov model (HMM) was structured to represent the transition of such histograms within the input sequence of depth maps. The likelihood provided by this HMM given a new sequence was used as a partial gait normality indicator. The second partial score was estimated by considering the change of pixel-based projections over a sequence of binary silhouettes. Differently from that study, our method directly embeds the transition of depth-related properties as well as implicitly considers the silhouette-based symmetry in a single processing flow.

## 3. Depth-Based Geometric Feature

**Figure 1:**
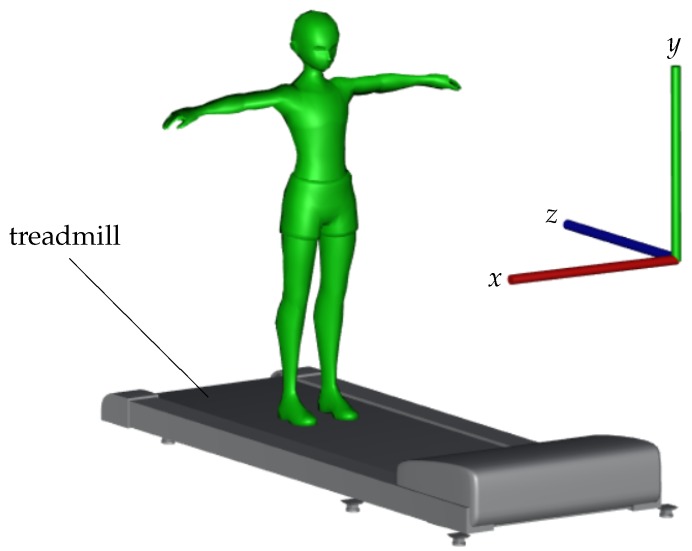
Our scene configuration and the corresponding 3D camera coordinate system.

The depth camera in our approach is placed in front of a person walking on a treadmill to capture depth frames from a frontal view. The right-handed camera coordinate system is thus, similar to the illustration in Figure 1, in which the positive *x* and *y* respectively point to the left and up while the *z*-axis points from the camera to the subject.

### 3.1. Human Body Segmentation

We only consider the body pixels, a step of background removal is thus, necessary since the input of the system is a sequence of depth maps. This operation can be easily performed by a depth-based segmentation (e.g., [11]). In detail, a 3D bounding box above the treadmill is specified and all points within this volume are considered belonging to human body. This method is appropriate for applications where the geometric relation between the camera and the treadmill is fixed. On the other hand, some SDKs provided together with the depth camera also have a functionality of human detection. For example, the Microsoft Kinect applies a random forest classifier on each depth map to assign a label (e.g., head, neck or shoulder) to each pixel, consequently the body is a collection of pixels of body-part labels [7,8]. We employ the latter method since it does not require a definition of a 3D region.

### 3.2. 3D Reprojection

Our geometric feature is computed in the 3D space, a reprojection is thus, performed to convert the depth body in each input frame to a 3D point cloud. Given a depth camera with an internal matrix of focal lengths $f_{x}$ and $f_{y}$, and principal point ${\left(c_{x},c_{y}\right)}^{⊤}$, a 3D point ${\left(x,y,z\right)}^{⊤}$ can be reconstructed from a 2D point ${\left(u,v\right)}^{⊤}$ with depth value *d* as

(1) $$x=\frac{(u-c_{x})d}{f_{x}},\;y=\frac{(v-c_{y})d}{f_{y}},\;z=d$$

There is an obvious advantage of using 3D point clouds instead of depth frames: the depth map is a projection result where each pixel contains 2D coordinates and a depth (u,v,d) while the reprojection maps the pixel into a point cloud in a uniform 3D coordinate system (x,y,z) which is more appropriate for geometric operations.

### 3.3. Cloud of Normal Vectors

Once a 3D point cloud is obtained for the body in each depth map, a collection of normal vectors is estimated to provide a raw representation of points’ direction. Surface normals are important properties of any geometric surface and are computed directly from the point cloud.

**Figure 2:**
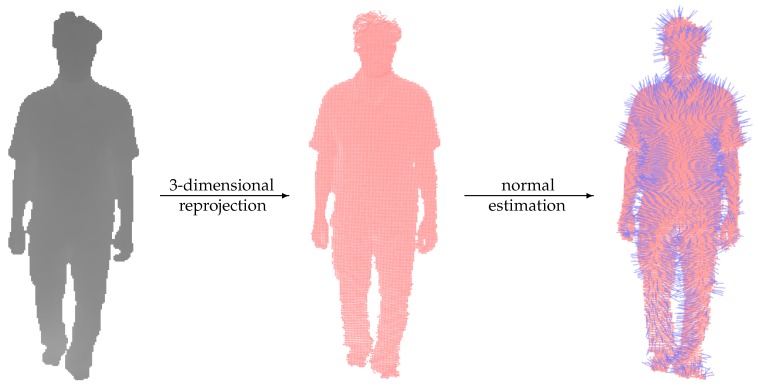
The normal cloud estimated from a depth map via a 3D reprojection. All normal vectors must point to the camera (i.e., non-positive *z*-coordinate) as a postprocessing of determined orientations.

The normal calculation of a 3D point is performed with eigenvectors and eigenvalues of a covariance matrix created from the points in its neighborhood [13]. Given an arbitrary 3D point cloud, such neighbor regions can be determined with the support of a KD-tree [14]. An illustration of this estimation is presented in Figure 2.

Let us notice that there is a trade-off between the processing time and the result of normal estimation. In detail, defining a neighborhood using a large radius leads to a longer processing time but the estimated normals are less sensitive to noise in the input point cloud. Recall that our approach directly applies on point clouds reconstructed from the depth maps without performing any enhancement (e.g., noise filtering). We defined a small neighborhood of 3-centimeters radius in our experiments (see Section 5) since normal vectors belonging to a large area will be further combined in the next step. This combination implicitly performs a noise removal and provides a reliable approximation of cloud surface direction.

### 3.4. Silhouette-Based Region Separation

A cloud of normal vectors cannot be directly used as a feature representing the human posture since different clouds might have various numbers of points. A possible solution is to segment the cloud into specific regions of interest. Concretely, we may define a fixed number of 3D regions together with their positions and combine the normal vectors within each one. This operation normalizes the cloud representation to a new array where each element corresponds to a 3D region.

**Figure 3:**
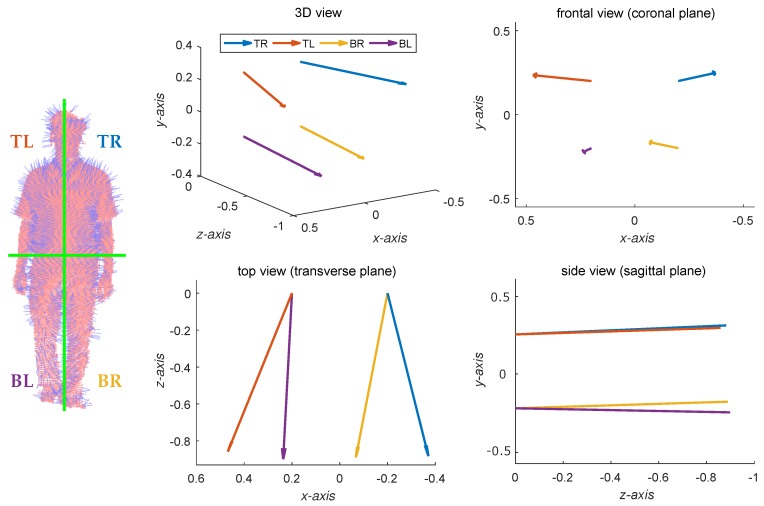
The separation applied on each cloud of normal vectors and the corresponding result of region-based accumulation. The frontal, side and top views are to provide an easy understanding for Section 3.5.

In our work, we separate a cloud of normal vectors according to human body anatomy and symmetry. Concretely, the body in each depth map is simply split into four equal-size regions using a 2×2 grid, in which the grid is easily determined as the bounding box of body silhouette. The vertical split is necessary for our objective: symmetry measurement. This split is expected to indicate the difference between the left and right body sides along the movement. The horizontal split is to individually consider the upper and lower body parts to assess whether using only the lower body is enough for the task of gait analysis (as done in [9,11]). Besides, this separation also reduces the risk of losing relevant information regarding specific upper and lower limb motion during walk. For example, when the left leg is moving forward during a stride, the left hand tends to be backward. The combination within each region (i.e., one of four grid cells) is simply performed as an algebraic addition of normal vectors. The representation of each input depth body is then a collection of four accumulated vectors $\left\{v_{\mathrm{TL}},v_{\mathrm{TR}},v_{\mathrm{BL}},v_{\mathrm{BR}}\right\}$ in the 3D camera space. An example of this step is shown in Figure 3, in which the terms TL, TR, BL, BR respectively indicate the top-left, top-right, bottom-left, and bottom-right regions. Although the resolution 2×2 is small, it represents well the human body anatomy typically used by physicians for localization for diagnosis or treatment and provided good results in our experiments in Section 5.

### 3.5. Angle Conversion

Instead of directly employing the four 3D vectors as a feature supporting the gait symmetry measurement, we convert them into a scalar value corresponding to the angle between the left and right side vectors projected onto a specific plane. In this work, we consider the three main planes used in anatomy and then evaluate their potential.

#### 3.5.1. Transverse Plane

**Figure 4:**
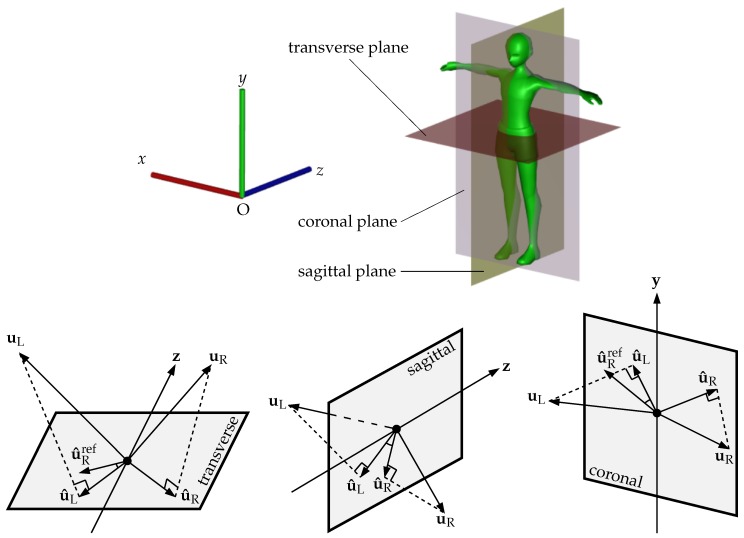
(**Top**) 3D representation of a body and the three anatomy planes together with the camera coordinate system. (**Bottom**) angle estimation based on transverse, sagittal and coronal planes. In the case that the coordinate system does not satisfy the requirement described in Figure 1, a rigid transformation is necessary to simplify the angle estimation.

The transverse plane vertically splits the body silhouette. Given the coordinate system shown in Figure 4, the plane is parallel with Oxz and goes through the horizontal line splitting the 2×2 grid (see Figure 3). Each 3D accumulated normal vector $u={\left(u_{x},u_{y},u_{z}\right)}^{⊤}$ is first projected onto the transverse plane to obtain a new vector $\hat{u}={\left(u_{x},0,u_{z}\right)}^{⊤}$. We then compare the direction of the vectors between the left and right sides. Assuming left-right symmetry, the mean value of the angle α between the left side vector ${\hat{u}}_{L}$ and the reflected right side vector ${\hat{u}}_{R}^{\mathrm{ref}}$ w.r.t. the positive *z* direction $z={\left(0,0,1\right)}^{⊤}$ should be small. This angle is computed as

(2) $$\alpha =\cos^{-1}\left(\frac{{\hat{u}}_{L}\cdot {\hat{u}}_{R}^{\mathrm{ref}}}{\|{\hat{u}}_{L}\left\|\cdot \right\|{\hat{u}}_{R}^{\mathrm{ref}}\|}\right)$$

with ${\hat{u}}_{R}^{\mathrm{ref}}=2\left(z\cdot {\hat{u}}_{R}\right)z-{\hat{u}}_{R}$ in which the dot notation · indicates the inner product.

#### 3.5.2. Sagittal Plane

The sagittal plane is parallel with the base plane Oyz and goes through the vertical line splitting the 2×2 grid. Similarly to the previous section, we also project each accumulated vector onto the sagittal plane and then calculate the angle between the left and right sides. In detail, the projected result of a vector $u={\left(u_{x},u_{y},u_{z}\right)}^{⊤}$ is determined as $\hat{u}={\left(0,u_{y},u_{z}\right)}^{⊤}$. The angle between ${\hat{u}}_{L}$ and ${\hat{u}}_{R}$ is determined as

(3) $$\beta =\cos^{-1}\left(\frac{{\hat{u}}_{L}\cdot {\hat{u}}_{R}}{\|{\hat{u}}_{L}\left\|\cdot \right\|{\hat{u}}_{R}\|}\right)$$

When extracting the angle feature using the sagittal plane, we do not need to reflect one of the two input vectors since the gait symmetry is analyzed via a comparison between left and right body parts instead of upper and lower ones (see Figure 4). During a normal (symmetric) walk, the mean value of ${\hat{u}}_{L}$ and ${\hat{u}}_{R}$ over a gait cycle should be close and the periodic angles β should be stable.

#### 3.5.3. Coronal Plane

The coronal plane is parallel with the base plane Oxy and is perpendicular to both transverse and sagittal planes. After projecting accumulated normal vectors onto the coronal plane, we perform the angle calculation in the same fashion as the transverse plane with a reflection w.r.t. the positive *y* direction $y={\left(0,1,0\right)}^{⊤}$ in order to indicate the left-right symmetry. Given an accumulated normal vector $u={\left(u_{x},u_{y},u_{z}\right)}^{⊤}$, its projection onto the coronal plane is thus, $\hat{u}={\left(u_{x},u_{y},0\right)}^{⊤}$ and the angle is computed as

(4) $$\gamma =\cos^{-1}\left(\frac{{\hat{u}}_{L}\cdot {\hat{u}}_{R}^{\mathrm{ref}}}{\|{\hat{u}}_{L}\left\|\cdot \right\|{\hat{u}}_{R}^{\mathrm{ref}}\|}\right)$$

where ${\hat{u}}_{R}^{\mathrm{ref}}=2\left(y\cdot {\hat{u}}_{R}\right)y-{\hat{u}}_{R}$.

#### 3.5.4. Feature Representation

After performing the feature extraction, each depth body is represented by three angles α, β and γ estimated for the lower and upper limbs. Recall that these three angles are independently processed in the next steps as well as in the experiments to give an evaluation. A geometric description of the three angle calculations is given in Figure 4 to provide a visual understanding.

## 4. Gait Symmetry Measurement

### 4.1. Basic Measurement

Given three angles α, β and γ estimated from a collection of accumulated normal vectors $v=\{v_{\mathrm{TL}},v_{\mathrm{TR}},v_{\mathrm{BL}},v_{\mathrm{BR}}\}$ (see Figure 3) according to the transverse, sagittal and coronal planes, our basic gait symmetry index is measured as the summation of top and bottom contributions, i.e.,

(5) $$\left\{\begin{array}{c} I_{\alpha }\left(v\right)=\alpha^{T}+\alpha^{B}\\ I_{\beta }\left(v\right)=\beta^{T}+\beta^{B}\\ I_{\gamma }\left(v\right)=\gamma^{T}+\gamma^{B} \end{array}\right.$$

The addition of non-negative angles corresponding to each anatomy plane allows the accumulation of the left-right differences resulting from the upper and lower limbs. We consider two different schemes for computing the mean value of **I** as our gait symmetry measurement.

### 4.2. Frame-Based Index

The first scheme is that a gait symmetry index is estimated for each depth body according to Equation (5). The temporal factor is then considered by calculating the average of these per-frame indices as the indicator of gait symmetry along the movement. In detail, given a sequence of *n* collections *v* of accumulated vectors measured from *n* consecutive depth bodies, the gait symmetry index corresponding to each of three angles α, β and γ is computed as

(6) $$I_{f}\left(v_{1..n}\right)=\frac{1}{n}\sum_{i=1}^{n}I\left(v_{i}\right)$$

where *n* should correspond to one or several gait cycles. During a normal symmetric walk, $I_{f}$ should be small for α and γ and stable for β over a gait cycle.

### 4.3. Segment-Based Index

Unlike the previous index measurement, the second scheme firstly performs an addition of accumulated normal vectors at the same grid cells over a sequence of consecutive depth bodies and then estimates the symmetry index using Equation (5) only once as

(7) $$I_{s}\left(v_{1..n}\right)=I\left(\frac{1}{n}\sum_{i=1}^{n}v_{i}\right)$$

This segment-based index may be interpreted as the within-frame measurement of a mean posture determined from a sequence of depth bodies. Therefore, for a symmetric gait, $I_{s}$ should be small for all three (sagittal, transverse and coronal) planes over a gait cycle.

## 5. Experiments

Since there is no benchmark dataset with ground truth gait symmetry index, we performed the evaluation of our method according to a specific application to distinguish normal and abnormal walking gaits. A good index measurement is expected to well separate the two gait types. In detail, the indices of normal walking gait provided by a symmetry indicator should be in a specific value range while the ones of anomalous gaits are distributed in other regions. These areas are expected to be well distinguishable. In practical applications, the gait symmetry index of a patient should converge from abnormal values to the normal range during a recovery. The measure of separation ability is the Area Under Curve (AUC) estimated according to the Receiver Operating Characteristic (ROC) curve since we are dealing with a problem of binary classification.

### 5.1. Dataset

Our experiments were performed on a dataset of multiple data types introduced in [15]. The dataset includes depth maps, point clouds, silhouettes and 3D skeletons that were acquired by a frontal depth camera. These data captured normal walking gaits and 8 abnormal ones that were performed by nine subjects (eight males, one female, 20–39 years old, 154–186 cm height and 51–95 kg mass) on a treadmill. These anomalous walking gaits were simulated by either padding a sole under one of the two feet or mounting a weight to an ankle. The former gaits were defined to focus on frontal asymmetry, i.e., the body centroid tends to be tilted to the left or right side, while the latter ones produce a movement with unequal speed and displacement between left and right legs. These gait abnormalities are appropriate to demonstrate the motion of patients having a problem with their lower limbs and have been used in related studies [11,16]. Each gait is represented by a sequence of 1200 frames corresponding to approximately 60 gait cycles. For this study, each of the 81 walking sequences was represented by 1200 consecutive depth maps in our evaluation.

This dataset was acquired by a Microsoft Kinect 2 at a frame rate of 13 fps. The camera was mounted at a height of 1.7 m. The distance between this camera and the subject was 2.3 m and the camera direction was parallel with the ground. Since Kinect two measures a depth map according to the Time-of-Flight technique, it provides smoother results with more details compared with cameras using structured light such as the Kinect version 1.

### 5.2. Evaluation Scheme

As mentioned in previous sections, we have different tests that can be independently performed to provide a comparison. First, there are three planes for the angle estimation including the transverse, sagittal and coronal ones. Second, we can individually consider the potential of frame-based and segment-based indices. Besides, we also evaluate the use of only the lower body (i.e., the angles estimated according to $v_{\mathrm{BL}}$ and $v_{\mathrm{BR}}$) since some recent studies (e.g., [9,11]) focused only on this part. Therefore, we obtained 12 evaluations for the 12 independent processing flows.

In order to provide an overall assessment for our method, we calculated an AUC over the entire dataset by focusing on a specific application: abnormal gait detection. Such evaluation assumes that the gait symmetry index belongs to a specific value range for normal walk while is beyond this range given an abnormal one. The leave-one-out cross-validation scheme was also employed to evaluate the distinguishing power of within-subject indices. Concretely, an AUC was calculated for each subject and the average AUC was finally used as the assessment result. This computation also allowed to compare our method with related studies where a training set is required.

### 5.3. Experimental Results

The AUCs obtained from the assessment of our symmetry index estimated on the whole body are shown in Table 1. It is obvious that the segment-based index in Equation (7) was more efficient for assessing gait symmetry than the one obtained by Equation (6) where the temporal factor was embedded after performing the index estimation for each frame. Table 1 also shows that using the sagittal-based angle provided better results than the transverse and coronal ones.

These properties are demonstrated again in Table 2 that presents the AUCs experimented on only the lower body. This table also shows that when the upper body was removed from the index estimation, the ability of gait symmetry assessment was reduced with decreasing of AUCs at corresponding positions in the table. Therefore, the whole body should be considered when dealing with problems related to gait symmetry index.

In order to provide a comparison with related studies, we reimplemented some methods as follows. The first selection was the HMM proposed in [9] that provides a gait normality index for each input sequence of skeletons. The second one was the MGCM introduced in [11] that computes the longitude difference between the left and right legs of an average aligned depth map as an indicator of gait asymmetry index. A common property between these two methods is the removal of upper body in the feature extraction stage. The model in [9] considered only 3D joints belonging to lower limbs while the one in [11] performed the estimation on a predefined leg zone. The third implemented approach was [12] where the researchers described the posture symmetry in both depth map and silhouette. These two features were independently extracted to give two scores. A combination of these scores was also proposed to improve the final gait index.

The AUCs measured according to the indices resulting from these three studies together with our best one (i.e., corresponding to the use of sagittal plane) are presented in Table 3. Each gait index indicating the overall gait symmetry was obtained by: a non-linear combination of per-cycle indices in [9], a direct consideration on the average gait cycle in [11], the per-segment mean index in [12], and our segment-based index described in Section 4.3.

Table 3 shows that the index given by our approach was better than the related studies since our indices of normal and abnormal walking gaits were more easily separable.

### 5.4. Combination of Gait Indices

As an attempt to improve the gait indices estimated according to the three planes, we performed weighted combinations that are expected to provide a better measure. Such combined gait index estimated on a sequence of *n* collections *v* of accumulated vectors is

(8) $$I\left(v_{1..n}\right)=w_{t}I_{t}\left(v_{1..n}\right)+w_{s}I_{s}\left(v_{1..n}\right)+w_{c}I_{c}\left(v_{1..n}\right)$$

where the subscripts *t*, *s* and *c* respectively indicate the transverse, sagittal and coronal planes. We can also combine only two of the three measures by simply assigning a zero weight to the remaining operand.

Instead of using a grid search to determine appropriate weights, we directly estimated them from the gait samples of the training set in the leave-one-out cross-validation scheme. The weight corresponding to each plane-related index was calculated as the variance of such index resulting from training samples of normal gaits. Variance was used here as a measure of information provided by each index. The abnormal gait patterns were not employed in this stage because the combination might be biased and would not work well in practical situations where various abnormal gaits may occur. We considered only segment-based gait index since it was better than the frame-based one (see Table 1 and Table 2). We observed that the combination of $I_{t}$ and $I_{s}$ significantly enhanced the gait index while the others (including the combination of all three measures) resulted in the same or slightly lower AUCs (see Table 4).

### 5.5. Discussion

According to the experimental results presented in the previous section, the following properties might be useful for further studies to extend the proposed approach as well as deal with the problem of gait index estimation.

First, the proposed method is appropriate to work on noisy depth maps as demonstrated by the experiments. Recall that there is no enhancement step (e.g., filtering) performed to improve the depth map quality. However, the number of such noisy (unreliable) points is much smaller compared with informative ones. By grouping the points into four large regions and then performing accumulation, the effect of noise can be significantly reduced. Another possible factor that could deform the body depth map is the subject’s clothing. Therefore, comfortable but tight fitting clothes should be worn during the examination.

Second, the temporal integration of depth features should be performed before applying Equation (5) to measure the symmetry. In other words, the use of segment-based index according to Equation (7) is preferred to the frame-based one. This means that surface normals of the mean posture over several gait cycles reveal important information about the gait symmetry. In summary, our index can be considered as an average measure of symmetry indices of consecutive gait cycles given a long sequence of depth maps.

Third, the upper body has its contribution in the gait analysis. When this body part does not participate in any stage, the symmetry description might lose useful information, and the resulting index is thus, less efficient.

Finally, the sagittal gait index provided consistently the best results on our dataset and played the main role in the improvement of combined indices. This result is supported by the literature where kinematic gait measurements in the sagittal plane provide the most appropriate information [17]. In addition, the time series of this index may be appropriate for further investigations on gait analysis such as gait cycle segmentation or gait event demarcation. Nevertheless, transverse and coronal planes’ measurements might provide additional information important in clinical gait analysis. Again, let us notice that employing these planes requires an appropriate setup of camera coordinate system as illustrated in Figure 1 in order to simplify consequent calculations.

Although the input of our method is a sequence of depth maps, the geometric feature is directly extracted from the corresponding 3D point clouds. Therefore, the proposed approach is still applicable given such cloud data. However, the stage of estimating normal vector for each 3D point might be slightly time-consuming (especially with high-density clouds) since it depends on the determination of point’s neighborhood. For example, when assigning a radius of 5 centimeters for neighborhood search, the processing time was 3 times longer than 3 centimeters while the system accuracy was almost unchanged. When the depth map is available, a pixel neighbor is useful for this task since we know the correspondence between a pixel and its reprojected 3D point. In summary, the computational cost might be unexpectedly changed depending on the input data, but this is not a significant problem since there are some algorithms supporting the fast calculation of normal vectors (e.g., [18,19]).

## 6. Conclusions

An original approach estimating a gait symmetry index from a sequence of depth maps has been presented in this paper. By employing a 3D reprojection, a geometric feature is proposed to extract useful posture characteristics according to the region-based accumulation of 3D normal vectors. Since surface normals are independent of the subject’s position on the treadmill, no registration of depth maps is required in our methodology. Two schemes of embedding temporal factor are also considered and evaluated to give a reasonable recommendation for further works. The potential of surface normals for gait asymmetry assessment has been demonstrated by experiments on a gait dataset of 97,200 depth maps where the obtained results outperformed related studies in the task of distinguishing normal and abnormal walking gaits. This vision-based approach is an alternative method for gait symmetry measurement beside conventional motion systems that employ wearable sensors. The practical advantages of our method are the use of low-price devices and its easy setup without run-time calibration (as approaches using multiple input signals) or accurate sensor placement. Our system may work as a patient screening tool providing relevant gait information during a treatment or recovery after surgery. In further works, possible extensions of the body surface normal features can be investigated to improve the gait symmetry index estimation. Besides, the proposed index will be measured on a larger dataset and the results will be collected over multiple sessions/days for assessing their stability as well as modeling their change during the recovery of patients. Finally, investigating partial surfaces according to body parts to provide limb-level motions could be an interesting extension.

## Figures and Tables

**Table 1:** AUCs obtained from experiments considering the whole body. The first and second highest results are emphasized in bold and underlined, respectively.

Test Data	Index Estimation	Transverse Plane	Sagittal Plane	Coronal Plane
All 9 subjects	frame-based	0.816	**0.870**	0.716
	segment-based	0.895	**0.966**	0.832
Leave-one-out	frame-based	0.819	**0.931**	0.722
	segment-based	0.903	**0.958**	0.819

**Table 2:** AUCs obtained from experiments considering only the lower body. The first and second highest results are emphasized in bold and underlined, respectively.

Test Data	Index Estimation	Transverse Plane	Sagittal Plane	Coronal Plane
All 9 subjects	frame-based	0.707	**0.727**	0.514
	segment-based	0.770	**0.949**	0.785
Leave-one-out	frame-based	0.722	**0.833**	0.500
	segment-based	0.806	**0.958**	0.819

**Table 3:** The AUCs of related studies that employ different input data types. The notations † and ‡ indicate the consideration of only lower body and the use of data augmentation, respectively. The best results are highlighted in bold.

Method	Input	All 9 Subjects	Leave-One-Out
HMM [9] ^†^	skeleton	-	0.778
MGCM [11] ${}^{†}$	depth map	0.830	0.875
HMM [12]	depth map ${}^{‡}$	-	0.569
Correlation [12]	silhouette	-	0.903
HMM + Correlation [12]	combination ${}^{‡}$	-	0.917
Ours (lower body) ${}^{†}$	depth map	0.949	0.958
Ours (full body)	depth map	**0.966**	**0.958**

**Table 4:** The AUCs estimated from each single gait index and possible combinations. The notations are similar to Equation (8) and the best results corresponding to each row are highlighted in bold.

	$I_{t}$	$I_{s}$	$I_{c}$	$I_{t}$ & $I_{s}$	$I_{s}$ & $I_{c}$	$I_{t}$ & $I_{c}$	$I_{t}$ & $I_{s}$ & $I_{c}$
Full body	0.903	0.958	0.819	**0.986**	0.972	0.903	**0.986**
Lower body	0.806	**0.958**	0.819	**0.958**	**0.958**	0.792	**0.958**

## Abbreviations

The following abbreviations are used in this manuscript:

AUC	Area Under Curve
ROC	Receiver Operating Characteristic
HMM	Hidden Markov Model
MGCM	Mean Gait Cycle Model
MDI	Mean Depth Image
